# KANPHOS: A Database of Kinase-Associated Neural Protein Phosphorylation in the Brain

**DOI:** 10.3390/cells11010047

**Published:** 2021-12-24

**Authors:** Rijwan Uddin Ahammad, Tomoki Nishioka, Junichiro Yoshimoto, Takayuki Kannon, Mutsuki Amano, Yasuhiro Funahashi, Daisuke Tsuboi, Md. Omar Faruk, Yukie Yamahashi, Kiyofumi Yamada, Taku Nagai, Kozo Kaibuchi

**Affiliations:** 1 Department of Cell Pharmacology, Graduate School of Medicine, Nagoya University, 65 Tsurumai, Nagoya 466-8550, Japan; rijwan.riju01@gmail.com (R.U.A.); m-amano@med.nagoya-u.ac.jp (M.A.); farukbmb@gmail.com (M.O.F.); 2 Institute for Comprehensive Medical Science, Fujita Health University, Toyoake 470-1192, Japan; tomoki.nishioka@fujita-hu.ac.jp (T.N.); yasuhiro.funahashi@fujita-hu.ac.jp (Y.F.); daisuke.tsuboi@fujita-hu.ac.jp (D.T.); yukie.yamahashi@fujita-hu.ac.jp (Y.Y.); 3 Division of Information Science, Graduate School of Science and Technology, Nara Institute of Science and Technology, Ikoma 630-0192, Japan; juniti-y@is.naist.jp; 4 Department of Bioinformatics and Genomics, Graduate School of Advanced Preventive Medical Science, Kanazawa University, Kanazawa 920-8640, Japan; kannon@med.kanazawa-u.ac.jp; 5 Department of Neuropsychopharmacology and Hospital Pharmacy, Graduate School of Medicine, Nagoya University, 65 Tsurumai, Nagoya 466-8550, Japan; kyamada@med.nagoya-u.ac.jp; 6 Division of Behavioral Neuropharmacology, International Center for Brain Science (ICBS), Fujita Health University, Toyoake 470-1192, Japan; taku.nagai@fujita-hu.ac.jp

**Keywords:** KANPHOS, phosphorylation, phosphoproteomics, signal transduction, molecular mechanism, adenosine signaling, neurodevelopmental disorders, intellectual disability

## Abstract

Protein phosphorylation plays critical roles in a variety of intracellular signaling pathways and physiological functions that are controlled by neurotransmitters and neuromodulators in the brain. Dysregulation of these signaling pathways has been implicated in neurodevelopmental disorders, including autism spectrum disorder, attention deficit hyperactivity disorder and schizophrenia. While recent advances in mass spectrometry-based proteomics have allowed us to identify approximately 280,000 phosphorylation sites, it remains largely unknown which sites are phosphorylated by which kinases. To overcome this issue, previously, we developed methods for comprehensive screening of the target substrates of given kinases, such as PKA and Rho-kinase, upon stimulation by extracellular signals and identified many candidate substrates for specific kinases and their phosphorylation sites. Here, we developed a novel online database to provide information about the phosphorylation signals identified by our methods, as well as those previously reported in the literature. The “KANPHOS” (Kinase-Associated Neural Phospho-Signaling) database and its web portal were built based on a next-generation XooNIps neuroinformatics tool. To explore the functionality of the KANPHOS database, we obtained phosphoproteomics data for adenosine-A2A-receptor signaling and its downstream MAPK-mediated signaling in the striatum/nucleus accumbens, registered them in KANPHOS, and analyzed the related pathways.

## 1. Introduction

Protein phosphorylation and dephosphorylation are critical post-translational modifications in the regulation of cell signaling networks, especially in the nervous system, where these mechanisms contribute to brain development, functions and disorders [1,2,3,4,5]. The levels of protein phosphorylation are regulated by the combined actions of protein kinases and phosphatases. Over 500 protein kinases are encoded in the human genome, and more than 280,000 phosphorylation sites have been characterized [6]. Protein phosphorylation events in the brain have been extensively studied since the early 1970s, and it has become apparent that nerve cells, like other cell types, are highly regulated by phosphorylation signals [7]. Dysregulation of these signaling pathways has been implicated in neurodevelopmental disorders, including autism spectrum disorder, attention deficit hyperactivity disorder and schizophrenia, as the therapeutic agents for these diseases are involved in phosphorylation signals. For example, the atypical antipsychotic agents risperidone and aripiprazole are used to treat exacerbations of autism spectrum disorder (ASD) [8,9], while chlorpromazine and haloperidol, typical antipsychotic agents, are utilized to treat schizophrenia [10,11]. These antipsychotic agents target the receptors of monoamines such as dopamine, serotonin and adrenaline, which are coupled to specific G protein-coupled receptors (GPCRs), and their downstream protein kinases [12,13,14]. Therefore, the identification of phosphorylated proteins and sites, together with the kinases and phosphatases responsible for regulating their phosphorylation, is very important for revealing cell signaling in the brain and finding associations with neurological disease pathogenesis. However, despite its importance, data describing phosphoproteins in the brain and their phosphorylation sites remain limited.

With the advancement of mass spectrometry (MS)-based proteomics approaches, a variety of databases have been developed to document experimentally verified in vivo and/or in vitro phosphorylation sites from numerous biological samples. Several phosphoproteomics databases have been developed, including UniProtKB [15], PhosphoELM [16], PhosphoSitePlus [17], PHOSIDA [18], SysPTM [19], jPOST [20], and Human Protein Reference Database (HPRD) [21]. However, none of these phosphoproteomics databases focus on brain phosphorylation events, and they fail to explain the upstream signaling events for phosphorylation because the identities of the kinases that phosphorylate the listed sites remain largely unknown. Many studies have attempted to predict the kinases responsible for phosphorylation at specific sites based on consensus amino acid sequence motifs [22,23,24], but these predictions are currently of little practical use because knowledge of the consensus sequence motifs for various kinases remains limited. To obtain kinase-oriented protein phosphorylation information, including data on phosphoproteins, phosphorylation sites and responsible kinases, we developed the kinase-interacting substrate screening (KISS) method for in vitro phosphoproteins [25,26,27] and the kinase-oriented substrate screening (KIOSS) method for in vivo phosphorylation signaling [26,27,28].

In this study, we developed and presented an online database named KANPHOS (Kinase-Associated Neural PHOspho-Signaling), (available online: https://kanphos.neuroinf.jp/ (accessed on 2 December 2021)) which provides phosphorylation signaling information, including data on phosphoproteins, phosphorylation sites and responsible kinases, in the brain. Moreover, we performed KIOSS to obtain novel phosphoproteomics data for adenosine-A2a-receptor signaling, including downstream MAPK-mediated signaling, in the striatum/nucleus accumbens and registered them in KANPHOS.

## 2. Materials and Methods

### 2.1. System Configuration and Application Software

The KANPHOS database is designed as a web-based database that is accessible from a web browser to allow simple and widespread use by researchers. It was built based on the neuroinformatics platform system named XooNIps (XOOPS module for Neuroinformatics) version 4.0 (RIKEN Brain science institute, Wako, Japan) [29]. XooNIps is an extended module of the extensible object-oriented portal system (XOOPS) (available online: https://xoops.org/ (accessed on 1 December 2021)), which is an OS-independent content management system (CMS) web application written in PHP (Hypertext Preprocessor, available online: https://www.php.net/ (accessed on 20 November 2021)). In KANPHOS, XooNIps runs on CentOS (available online: https://www.centos.org/ (accessed on 1 December 2021)) using Apache (The Apache HTTP Server Project, available online: https://httpd.apache.org/ (accessed on 21 November 2021)) as the web server and MySQL (available online: https://www.mysql.com/ (accessed on 24 November 2021)) as the database management system.

The stable operation and updating of web-based databases requires substantial familiarity with web and network systems. To solve these problems, CMSs for the construction and management of web-based databases have become popular. The database we sought to develop needed to be able to handle a very large and continuously increasing amount of experimental data on protein kinases and phosphorylation sites. XooNIps is an easy-to-use software that can facilitate the construction of web-based databases for organizing vast amounts of information with metadata in various electronic formats and sharing it with users around the world on the internet.

### 2.2. Data Collection, Management and Analysis Tool

As of October 2021, approximately 10,000 records have been registered in KANPHOS. Each record is composed of a pair, a protein phosphorylation site and its modifier (i.e., the corresponding kinase or extracellular signal, such as a neurotransmitter). Of these, 6228 records were identified based on our experimental results, while 3736 records were based on publications from other groups. To facilitate analysis, KANPHOS supports three search modes: (1) search for substrates phosphorylated by a specific kinase; (2) search for kinases phosphorylating a specific protein; and (3) search for kinases and their target substrates in a specific signaling pathway. All proteins registered in KANPHOS are associated with a UniProt ID to facilitate linkage with 12 external databases (Table S1). In particular, protein associations with Gene Ontology (GO) terms and KEGG pathways are useful for exploring potential as yet unidentified functions.

### 2.3. Preparation and Incubation of Coronal Slices

Coronal slices were prepared from male C57BL/6 mice as described previously [30]. In our adenosine and MAPK experiment, we try to minimize the number mice sacrifice. For each experiment, one male C57BL/6 mice (total 6) were sacrificed humanely by decapitation. From one mouse, we dissected striatal/accumbal slices and distributed them into 3 or 4 treatment conditions. The brain was removed and kept in Krebs-HCO_3_^−^ buffer (124 mM NaCl, 4 mM KCl, 26 mM NaHCO_3_, 1.5 mM CaCl_2_, 1.25 mM KH_2_PO_4_, 1.5 mM MgSO_4_, and 10 mM D-glucose, pH 7.4). Coronal brain slices (350 μm) were prepared using a VT1200S vibratome (Leica Microsystems, Wetzlar, Germany). After dissecting of the whole striatum/NAc, coronal slices were incubated at 30 °C in Krebs-HCO_3_^−^ buffer with 10 μg/mL adenosine deaminase (Roche, Basel, Switzerland) for 30 min under constant oxygenation with 95% O_2_/5% CO_2_. The buffer was replaced with fresh Krebs-HCO_3_^−^ buffer and preincubated for 30 min. Coronal slices were pretreated with the indicated inhibitors (quinpirole 1 μM for 10 min, U0126 10 μM for 30 min) and then stimulated with the indicated activators (CGS21680 5 μM for 5 min, okadaic acid 1 μM for 60 min). After stimulation, slices were immediately frozen in liquid nitrogen and stored at −80 °C. The slices were lysed in lysis buffer (1% SDS, 1 mM EDTA, 1 mM DTT, phosphatase inhibitor cocktail (Roche, Indianapolis, IN, USA), protease inhibitor cocktail (Roche), pH 7.0) and sonicated for 20 s. The protein concentration of lysates was determined by BCA assay (FUJIFILM Wako). The A2AR-specific agonist CGS21680 (Cat No: Sigma-Aldrich Co., St. Louis, MO, USA), D2R-specific agonist quinpirole (Cat No., 1061; TOCRIS Bioscience, Bristol, UK), phosphatase inhibitor okadaic acid (OA) (primarily a PP2A inhibitor) (Wako Chemicals, Osaka, Japan), and MEK1/2 inhibitor U0126 (Cat No., 1144; TOCRIS Bioscience, Bristol, UK) were used for slice culture treatment.

### 2.4. Mass Spectrometry

Mass spectrometry (MS) was performed as previously described [26] with some modifications. Frozen mouse brain slices were lysed in lysis buffer (1 mM EDTA, 50 mM Tris/HCl, 150 mM NaCl, 0.5% sodium deoxycholate, 1% NP-40, 0.1% SDS, PhosStop (Roche), and protease inhibitor cocktail (Roche), pH 7.5) and sonicated for 15 s. Supernatant was collected after centrifugation of lysate at 15,000 rpm for 10 min at 4 °C. Protein concentrations of lysates were equalized before incubation with GST-14-3-3*ζ* (500 pmol) immobilized on glutathione sepharose 4B beads (GE Healthcare Bio-Sciences AB, Uppsala, Sweden) for 60 min at 4 °C. The beads were washed with lysis buffer three times, followed by wash with wash buffer (1 mM EDTA, 50 mM Tris/HCl and150 mM NaCl, pH 7.5) three times. Using guanidine solution (7 M guanidine and 50 mM Tris), bound proteins were removed from the beads, followed by reduction with 5 mM dithiothreitol for 30 min, and alkylation by10 mM iodoacetamide for 1 h at dark. The proteins were digested with Trypsin/Lys-C (Promega) and incubated overnight at 37 °C. Demineralization was performed using SPE c-tips (Nikkyo Technos, Tokyo, Japan) and phosphopeptide was enriched by TiO_2_ column (Titansphere^®^ Phos-TiO kit, GL Sciences, Tokyo, Japan) according to the manufacturer’s instructions. Finally, all the peptides were analyzed by liquid chromatography/mass spectrometry (LC/MS) using an Orbitrap Fusion mass spectrometer (Thermo Fisher Scientific Inc., Waltham, MA, USA), which was coupled with an UltiMate3000 RSLCnano LC system (Dionex Co., Amsterdam, The Netherlands) using a nano High-performance liquid chromatography (HPLC) capillary column, C18, 3 µm, 150 mm × 75 µm (Nikkyo Technos Co., Tokyo, Japan) via a nanoelectrospray ion source. Reversed-phase chromatography was performed with solvent A (2% acetonitrile, 0.1% formic acid) and solvent B (95% acetonitrile, 0.1% formic acid) with a linear gradient (0 min, 5% B; 100 min, 40% B) at a flow rate of 300 nL/min. Prior to MS/MS analysis, a precursor ion scan was carried out using a 400–1600 mass to charge ratio (*m*/*z*) and then MS/MS analysis was performed by isolation at 0.8 Th with the quadrupole, HCD fragmentation with a normalized collision energy of 30%, and rapid scan MS analysis in the ion trap. Precursor ions with charge states of 2–6 were sampled for MS2 only. The instrument was run in a top speed mode with 3 s per cycle. Phosphoprotein identification was performed by MaxQuant software (version 1.4.1.2) (Max-Planck-Institute of Biochemistry, Computational Systems Biochemistry, Planegg, Germany) against the reference *Mus musculus* proteome database obtained from UniProtKB (release in November 2018) (accessed on 4 June 2021) [31]. We set carbamidomethylation of cysteine as a fixed modification, whereas methionine oxidation, Ser/Thr/Tyr phosphorylation, and N-terminal acetylation were set as variable modifications. False discovery rates (FDRs) for the peptide, protein, and site levels were set to 0.01. The ion peak intensities obtained from three independent experiments were analyzed. Gene Ontology (GO) analysis (biological process) and KEGG pathway analysis were performed using DAVID bioinformatics resources (available online: https://david.ncifcrf.gov/ (accessed on 7 November 2021)) and Reactome analysis (available online: http://www.reactome.org/, accessed on 7 November 2021).

### 2.5. SDS-PAGE and Immunoblotting

The proteins were separated via SDS-PAGE using 8% polyacrylamide gels (Nacalai Tesque, Kyoto, Japan) and transferred to polyvinylidene difluoride membranes (Immobilon-FL, Merck). The membranes were blocked for 30 min at room temperature with Blocking-One (Nacalai Tesque) and then incubated with primary antibody for overnight at 4 °C. Following primary antibodies were used: rabbit monoclonal anti-phospho-GluR1 (S845) (RRID: AB_10860773) from CST; mouse monoclonal anti-GluR1 (RRID: AB_11212678) from EMD Millipore; rabbit monoclonal anti-phospho-p44/42 MAPK (ERK1/2) (T202/Y204) (RRID: AB_2315112), mouse monoclonal anti-p44/42 MAPK (ERK1/2) (RRID: AB_10695739) from CST; anti-phospho-Rap1gap (S563) [32], rabbit anti-Rap1gap (RRID: AB_777621) from Abcam (Cambridge, UK), anti-phospho-Rasgrp2 (S116/117) [26], and rabbit polyclonal anti-Rasgrp2 (Abcam Cat# ab126039, RRID:AB_11128113). Membranes were washed with Tris-buffered saline (TBST; 20 mM Tris, 150 mM NaCl, 0.05% Tween 20, pH 7.6) three times and incubated with secondary antibodies: goat anti-mouse IgG, Alexa Fluor 680 (RRID: AB_2535724, Thermo Fisher Scientific), goat anti-rabbit IgG Alexa Fluor 680 (RRID: AB_2535758, Thermo Fisher Scientific), or anti-mouse IgG (H+L) DyLight 800 conjugate (RRID: AB_10693543, Cell Signaling Technology) at room temperature for 30 min. Specific binding of proteins was detected using an infrared imaging system (LI-COR Biosciences, Lincoln, NE, USA) and band intensities were quantified by Image Studio software (version 3.1.4) (RRID: SCR_015795, LI-COR Biosciences, Lincoln, NE, USA).

## 3. Results

### 3.1. Construction and Content of the KANPHOS Database

**Figure 1:**
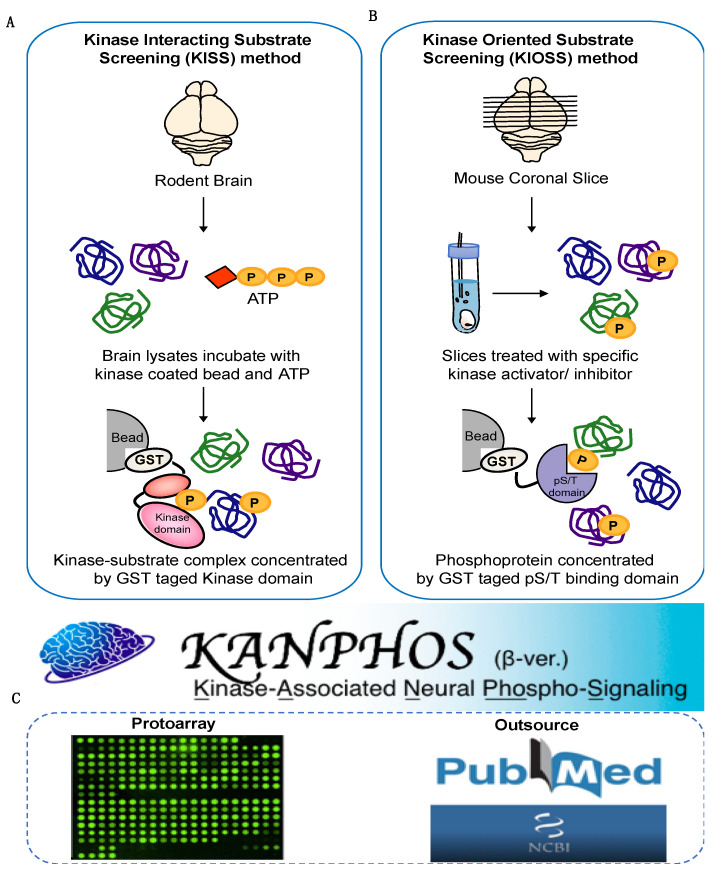
The schema for the construction of KANPHOS. (**A**) Schematic representation of the Kinase-Interacting Substrate Screening (KISS). In KISS method, rodent brain lysates were extracted and incubated with specific kinase-tag beads and ATP. The bound proteins were subjected to liquid chromatography tandem mass spectrometry (LC-MS/MS) to identify the phosphorylated proteins and their phosphorylation sites. (**B**) Schematic representation of the Kinase-Oriented Substrate Screening (KIOSS) method. In KIOSS method, mouse coronal brain slices were treated with specific kinase activator/receptor agonist or kinase inhibitor/receptor antagonist to stimulate a specific signaling pathway and then incubated with GST tag pS/T binding domain (such as 14-3-3ζ) to enrich phosphoproteins. The bound proteins were subjected to LC-MS/MS to identify the phosphorylated proteins and their phosphorylation sites. (**C**) In addition to our own developed phosphoproteomic method, data were also manually curated from the literature and protoarray.

**Figure 2:**
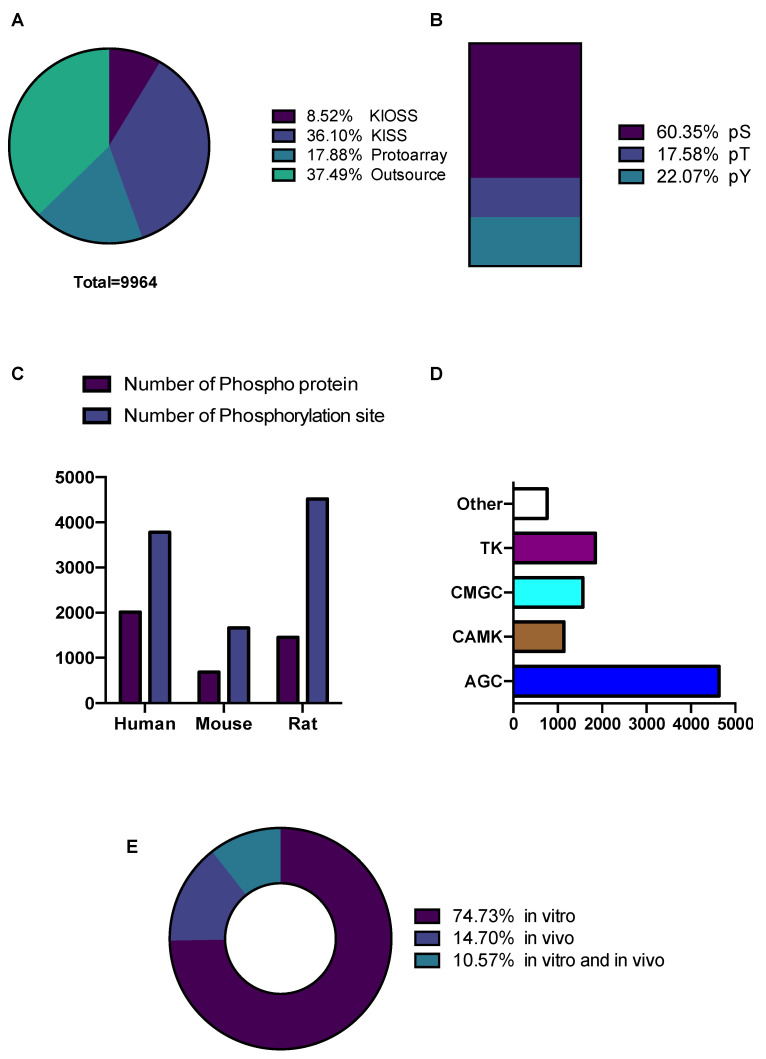
Data statistics and content of KANPHOS database. (**A**) Number of phosphorylation sites that were enlisted in KANPHOS using different methods; (**B**) Percentage of phosphoserine (pS), phosphothreonine (pT), and phosphotyrosine (pY) distribution; (**C**) Number of Phosphoprotein and phosphorylation sites identified in different species; (**D**) Distribution of phosphorylation sites among kinase families; (**E**) Percentage of phosphorylation sites identified in vitro, in vivo, and both in vitro and in vivo.

To construct the KANPHOS database of protein phosphorylation modification in the brain, we integrated data mainly from our own developed phosphoproteomic method (45%), from the literature (37%) and from Protoarray (18%) (Figure 1 and Figure 2A). To obtain kinase-oriented protein phosphorylation information, we developed the KISS method for in vitro phosphoproteins [33] and the KIOSS approach for in vivo phosphorylation dependent signaling [34]. In the KISS method, an active catalytic domain of the kinase of interest is used as bait to enrich the substrates, and the interacting proteins were subsequently phosphorylated by the active catalytic domain by incubation with or without ATP (Figure 1A). Liquid chromatography/tandem mass spectrometry (LC/MS/MS) phosphoproteomics was performed to identify the kinase-interacting proteins (candidate substrates) and their phosphorylation sites. We applied the KISS approach to a wide range of Ser/Thr and Tyr protein kinase families: Rho-kinase, Protein Kinase A (PKA), Protein Kinase B (PKB or Akt), protein kinase N (PKN), calcium/calmodulin-dependent protein kinase I (CaMK1), mitogen-activated protein kinases (MAPK), cyclin-dependent kinase 5 (CDK5), p21 (RAC1) activated kinase 7 (PAK7), Glycogen Synthase Kinase 3 Beta (GSK3β), Casein kinase 2 (CK2), Lck/Yes-related novel protein tyrosine kinase (LYN) and FYN oncogenes related to SRC, FGR, and YES (FYN) (Table 1) [25,35,36].

In the KIOSS method, cell or tissue cultures were treated with an agonist, kinase inhibitor, and/or phosphatase inhibitor, and phosphoproteins were enriched from the lysate with phospho-Ser/Thr-binding proteins, followed by LC-MS/MS analyses to identify phosphoproteins and their phosphorylation sites (Figure 1B). We chose the 14-3-3*ζ* protein, tryptophan–tryptophan (WW) domain, and forkhead-associated (FHA) domain as the phospho-Ser/Thr-binding modules because these modules contain proteins that broadly regulate the function of their target proteins in a phosphorylation state-dependent manner and contribute to cellular phospho-signaling networks [37]. We performed KIOSS to analyze Rho-kinase-mediated phosphorylation in HeLa cells [27], PKA and dopamine D1 receptor-mediated phosphorylation in the mouse striatum [26]. The ratio of protein phosphorylation sites identified using the KISS method was 36%, whereas the KIOSS method identified 9% of the total entries (Figure 2A).

Published phosphoproteomics datasets were collected from the literature by searching PubMed using keywords including ‘phosphoproteomic’, ‘phosphoproteomics’ and ‘phosphoproteome’. The retrieved literature was manually reviewed and curated by our professional staff; experimentally identified candidate substrates and phosphorylation site information were collected and cross-checked with other databases, such as UniProt, PhosphositePlus, and PhosphoELM. In addition to manual curation from the literature, data were also collected from Protoarray, which provides data generated by substrate screening for proteins phosphorylated by recombinant kinases. Each kinase was assayed at 5 nM and 50 nM in the presence of γ33P ATP to explore the phosphorylation of more than 9000 human proteins printed on the Protoarray^®^ microarray (Kinase Substrate Identification service, Invitrogen) [38,39]. However, these data did not contain information relating to protein phosphorylation sites. The ratio of the protein phosphorylation identified from the published literature and the Protoarray method was 37% and 18% of the total entries, respectively (Figure 2A).

The distribution of phosphoproteins and their phosphorylation sites, different residue types, and species are summarized and represented in Figure 2. In total, 9964 phosphorylation sites were identified in 4141 phosphoproteins. The numbers (and percentages) of phosphoserine (pS), phosphothreonine (pT), and phosphotyrosine (pY) were 4937 (60%), 1438 (18%), and 1850 (22%), respectively (Figure 2B); a higher ratio of pY was detected because of data obtained from FYN and LYN kinase using the KISS method. Most of the identified phosphorylation sites were phosphoserine. Although data from different species were included in KANPHOS, most of the phosphorylation events listed here are from rodents. The three species with the highest levels of phosphoproteins (phosphorylation sites) were humans (2010 (3782)), rats (1452 (4520)), and mice (680 (1662)) (Figure 2C). Furthermore, the potential upstream kinases associated with these phosphorylation sites were annotated using kinase motif preferences (Figure 2D). Most of the predicted phosphorylation sites belonged to the AGC family kinase (4631). The other kinase families, CAMK, CMGC, and TK, contain 1141, 1564 and 1850 phosphorylation sites, respectively. Among the predicted phosphorylation sites, 7445 (75%) were detected in vitro, 1465 (15%) were detected in vivo and approximately 1053 (10%) were detected both in vitro and in vivo (Figure 2E).

### 3.2. Data Accessibility and KANPHOS Workflow

**Figure 3:**
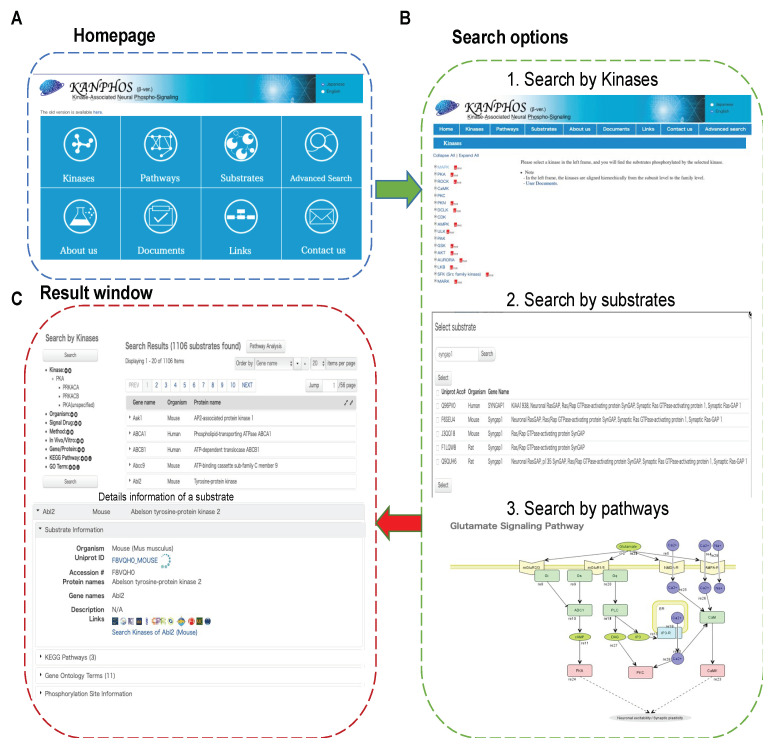
Overview of the KANPHOS workflow. (**A**) Homepage and browse function; (**B**) Simple search options (1) Search by kinases,(2) Search by substrates, and (3) Search by pathways; (**C**) Result window containing information about the phosphorylation sites, possible upstream signal and kinase.

To facilitate access to the KANPHOS database in a user-friendly manner, a web interface was developed with browsing and search options to facilitate the query of protein phosphorylation events. An overview of the KANPHOS workflow is shown in Figure 3. Three search options were provided on the KANPHOS homepage: 1. search for the proteins phosphorylated by selected kinases; 2. search for the kinase responsible for phosphorylating selected substrates; and 3. search for phosphorylated proteins along with responsible kinase within pathway maps. Advanced search options are available to allow search by gene symbol/protein names, species, KEGG pathways, and Gene Ontology in a window at the left of the results list (Figure S1). The search results can be further filtered by experimental conditions, methods, extracellular signals, in vivo/in vitro, and chemical treatments (Figure S1). When advanced search options and/or experimental conditions are selected, only the candidate substrates that contain the string that is entered will be displayed in the search results, which enables users to quickly retrieve their data of interest. In the search results page, the results are listed, and detailed protein information can be shown by clicking the protein names. Under the selected protein, general information about the proteins, associated pathways, annotated GO terms, and phosphorylation sites can be seen on the same page. Small icons that directly link to the protein data in HUGO, rat genome database (RGD), mouse genome informatics (MGI) database, GeneCards, Allen Brain Atlas, HomoloGene, HuGENavigator, H-inv, MalaCards, and Diseases are displayed next to the general information (Table S1). Users can click the phosphorylation site information option to view the detailed information, which includes the responsible kinases, phosphorylation positions and residue types, peptide sequences, experimental methods, extracellular signals and associated drug treatment (Figure S2).

### 3.3. Case Study: Identification of Adenosine-A2A Receptor Signaling and MAPK-Mediated Signaling Molecules Using KANPHOS

To examine how the KANPHOS database works, we performed KIOSS to obtain phosphoproteomic data downstream of the adenosine signaling pathway in the striatum/NAc. We used this study as a model to demonstrate the ability of the KANPHOS database to identify novel phosphorylation-dependent signaling dynamics in the brain.

**Figure 4:**
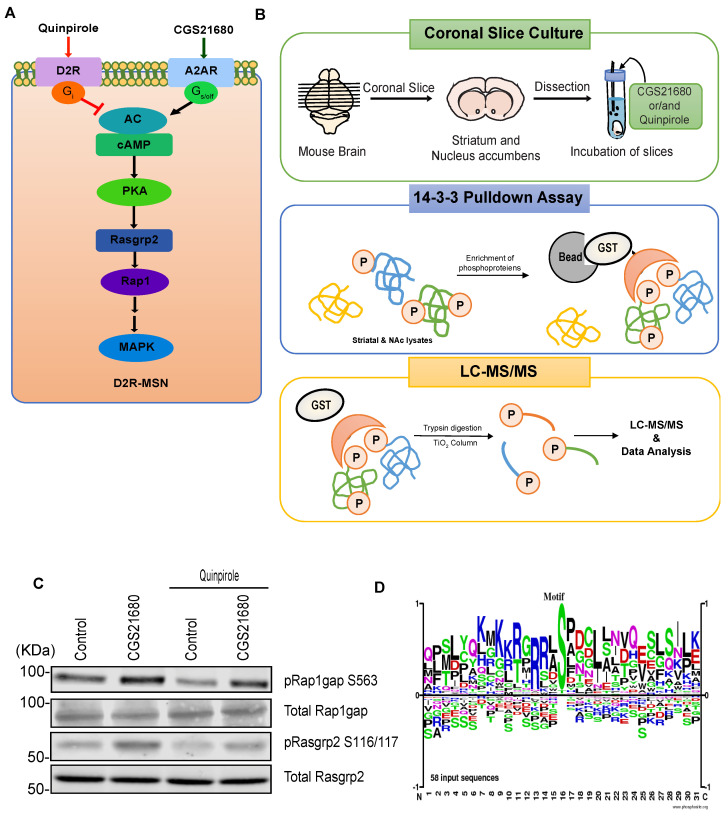
Identification of signaling molecules downstream of Adenosine-A2AR pathway in striatum/NAc. (**A**) Model of A2AR signaling pathway in D2R-MSN; (**B**) Scheme for the phosphoproteomic analysis used to identify candidate substrates downstream of the A2AR pathway. Striatal/accumbal slices were stimulated with CGS21680 (5 μM) for 5 min after pretreatment with quinpirole (1 μM) for 10 min to induce A2AR-mediated phosphorylation of the phosphoprotein and then subjected to 14-3-3ζ affinity chromatography followed by LC-MS/MS; (**C**) Stimulation of adenosine A2AR promotes PKA-mediated phosphorylation of Rap1gap, and Rasgrp2 in striatal/accumbal slices; (**D**) Motif analysis of the identified phosphopeptides downstream of A2AR signaling is shown.

Adenosine is an important neuromodulator in the striatum/NAc that acts through adenosine A1 receptor (A1R) or adenosine A2A receptor (A2AR) [40]. Approximately 95% of striatal and accumbal neurons are medium spiny neurons (MSNs) that express either the dopamine D1 receptor (D1R-MSNs) or dopamine D2 receptor (D2R-MSNs) [41,42]. In the striatum/NAc, D2R-MSNs express A2AR, which couples to the G_olf_ protein, whereas D1R-MSNs express A1R, which couples to the G_i_ protein [43,44,45]. Thus, PKA activity in D2R-MSNs was positively regulated by adenosine-A2AR and negatively regulated by dopamine-D2R (Figure 4A). To identify novel phosphoproteins downstream of adenosine-A2AR signaling, mouse striatum/NAc slices were treated with the A2AR agonist CGS21680 and the D2R agonist quinpirole (Figure 4B). PKA activity was confirmed by monitoring the phosphorylation level of well-known PKA substrates (Rap1gap at S563, and Rasgrp2 at S116/117) by immunoblotting [46,47]. Treatment of slices with CGS21680 increased the phosphorylation level of Rap1gap at S563, and Rasgrp2 at S116/117 (Figure 4C). The CGS21680-stimulated phosphorylation of Rap1gap at S563, and Rasgrp2 at S116/117 was blocked by pretreatment with quinpirole (Figure 4C). These results suggest that adenosine A2AR regulates PKA activity in the striatum and NAc.

Next, we performed phosphoproteomic analysis using the KIOSS method, in which affinity beads coated with the pS/T-binding domain 14-3-3ζ were used to enrich phosphorylated proteins. The bound proteins were digested using trypsin/Lys-C and loaded onto a TiO_2_ column to enrich for phosphopeptides, followed by LC-MS/MS to identify the phosphorylated proteins and their phosphorylation sites (Figure 4B). Almost 80% of the phosphoproteins enhanced by A2AR agonists were inhibited by D2R agonists; thus, these phosphoproteins represent candidate substrates of adenosine signaling. These candidates were listed in Table 2. Motif analysis using the amino acid sequence logo of the identified phosphopeptides revealed that approximately 55% of the phosphorylation sites contained basic residues at the -3 and -2 positions, suggesting that the AGC group of protein kinase such as PKA was responsible for these phosphorylation events (Figure 4D). Proline at the +1 position was observed at about 20% of these sites, suggesting that they are phosphorylation sites for proline-oriented kinases such as MAPK1/3.

**Figure 5:**
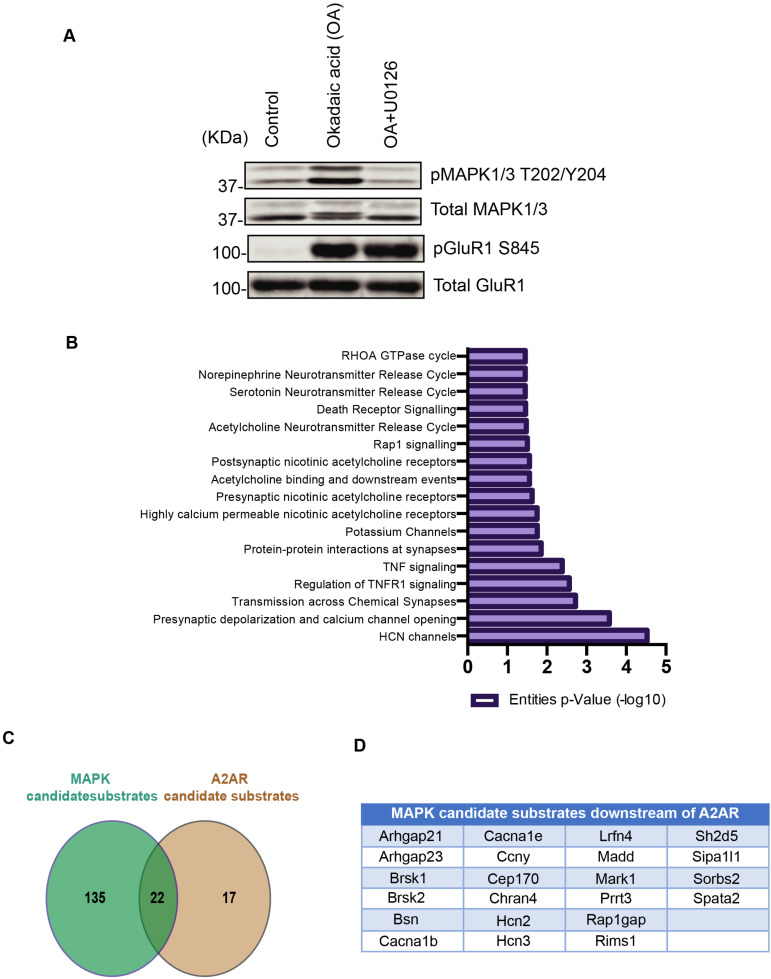
Identification of MAPK candidate substrates downstream of A2AR signaling in striatum/NAc. (**A**) Screening of phosphoproteins downstream of MAPK-mediated signaling. Striatal/accumbal slices were treated with okadaic acid (1 μM) for 60 min after pretreatment of MEK inhibitor U0126 (10 µM) for 30 min. OA induced the phosphorylation ERK at T202/Y204 and PKA substrate GluR1 at S845, whereas the pretreatment of MEK inhibitor reduced the phosphorylation of ERK but had no effect on PKA substrate GluR1 phosphorylation; (**B**) In silico analysis of the identified MAPK candidate substrates using the Reactome database (available online: http://www.reactome.org/ (accessed on 7 November 2021)) is shown; (**C**) Overlapping of MAPK and A2AR candidate substrates; (**D**) List of MAPK candidate substrates downstream of A2AR signaling.

We previously found that adenosine activated Rap1 through the phosphorylation of Rap1gap and Rasgrp2, thereby activating the MEK-MAPK pathway, which plays a critical role in adenosine-mediated aversive learning [48]. We next performed the KIOSS approach to explore MAPK-mediated signaling. The striatum/NAc slices were treated with a phosphatase inhibitor (okadaic acid) and/or a MEK inhibitor (U0126). Treatment of the slices with okadaic acid increased the phosphorylation of MAPK1/3 at T202/Y204, whereas pretreatment with a MEK inhibitor (U0126) reduced this okadaic acid mediated phosphorylation (Figure 5A). Treatment with okadaic acid also increased the phosphorylation of known PKA substrate GluR1 at S845, but pretreatment with U0126 did not affect this phosphorylation, indicating that U0126 specifically inhibited MAPK1/3 activation. The cell lysates were pulled down by beads coated with 14-3-3ζ to enrich for phosphoproteins. The bound proteins were subjected to tryptic digestion and LC-MS/MS to identify the phosphorylated proteins and their phosphorylation sites. The okadaic acid treatment induced the phosphorylation of huge numbers of proteins, including MAPK substrates, and pretreatment with U0126 specifically inhibited MAPK-mediated phosphorylation. Thus, the comparison between the treatments with okadaic acid and okadaic acid with U0126 identified more than hundreds of MAPK candidate substrates (Table 3). Comparison of MAPK candidate substrates with A2AR downstream phosphoproteins identified 22 phosphoproteins that were phosphorylated by MAPK downstream of adenosine signaling (Figure 5C,D). There were more candidate substrates obtained by the KIOSS method than those phosphorylated by MAPK downstream of the A2AR agonist. The candidate substrates did not overlap much. This may be due to the higher sensitivity of the KIOSS method. The identification of the related signaling pathways to the obtained phosphoproteins by using the Reactome database (available online: http://www.reactome.org/ (accessed on 7 November 2021)) revealed several potassium and Hyperpolarization-activated cyclic nucleotide gated (HCN) channels that may potentially be involved in neuronal excitability (Figure 5B).

**Figure 6:**
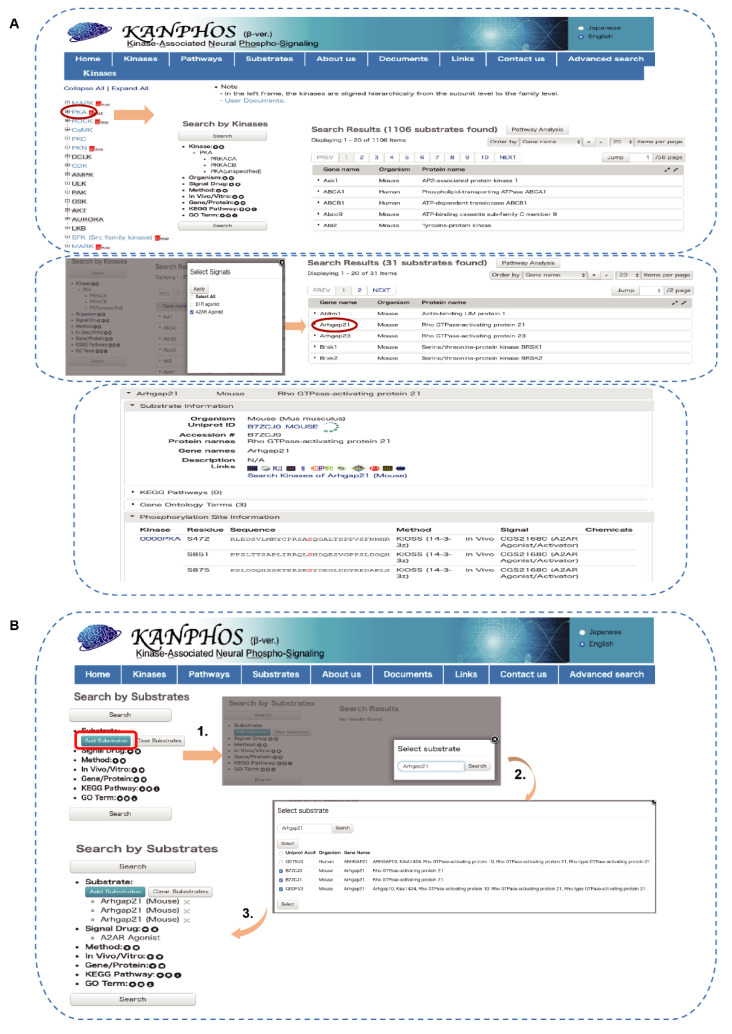
Workflow of KANPHOS for the identification of adenosine-A2A receptor-mediated signaling molecules. (**A**) Identification of Arhgap21 as a PKA candidate substrate downstream of adenosine signaling using “Kinases” search. Red circle represents the selected option and arrow indicates result outcome after each selection; (**B**) Identification of kinases which may phosphorylate Arhgap21 downstream of adenosine signaling using “Substrates” search. Red rectangle represents the selected search option and arrow indicates result outcome after each selection.

Among the identified phosphoproteins, Arhgap21 was detected as a candidate substrate for both PKA and MAPK downstream of adenosine signaling. In the next section, we describe the use of the KANPHOS database using Arhgap21 as an example. Phosphorylated proteins can be searched by clicking the “Kinases” icon on the home page, which will open the “kinases” page. Protein kinases are listed on the left of the page, and subtype cascades can be opened by clicking the “plus” icon associated with each kinase (Figure 6A). There are links to the IUPHAR (International union of basic and clinical pharmacology) protein kinase pages for each kinase. As we previously described, adenosine signaling activates PKA; therefore, if we choose PKA from the kinase list, the result page will be open. Using the advanced search window on the left side of the result page, we can further filter the candidate substrates list by selecting “Signal drug” (A2AR agonist) to identify phosphoproteins downstream of adenosine signaling. A list of candidate phosphoproteins will appear, listing only perfect matches to the string that was entered. From the list of candidate substrates, we chose Arhgap21 and clicked on it to obtain general information about the protein and detailed information about the phosphorylation sites (Figure 6A).

To identify the kinase responsible for phosphorylating a specific substrate, the user can click on “Substrates” icons on the home page and then enter the substrate name (Figure 6B). Here, we entered Arhgap21 and then clicked the “Search” button to generate a list of available substrate of Arhgap21 in various species. We then selected only those substrate data that were obtained from mice and clicked the “Select” button to add the substrate name. This opened a list of kinases that phosphorylate Arhgap21 in mice. A similar filter option was applied in the advanced search option “Signal drug” (A2AR agonist) to identify kinases (PKA, MAPK) that phosphorylate Arhgap21 in adenosine signaling (Figure 6B).

### 3.4. Pathway Analysis Using KANPHOS Revealed That HCN and Calcium Channel Proteins Are Involved in the MEK-MAPK Pathway Downstream of A2AR

**Figure 7:**
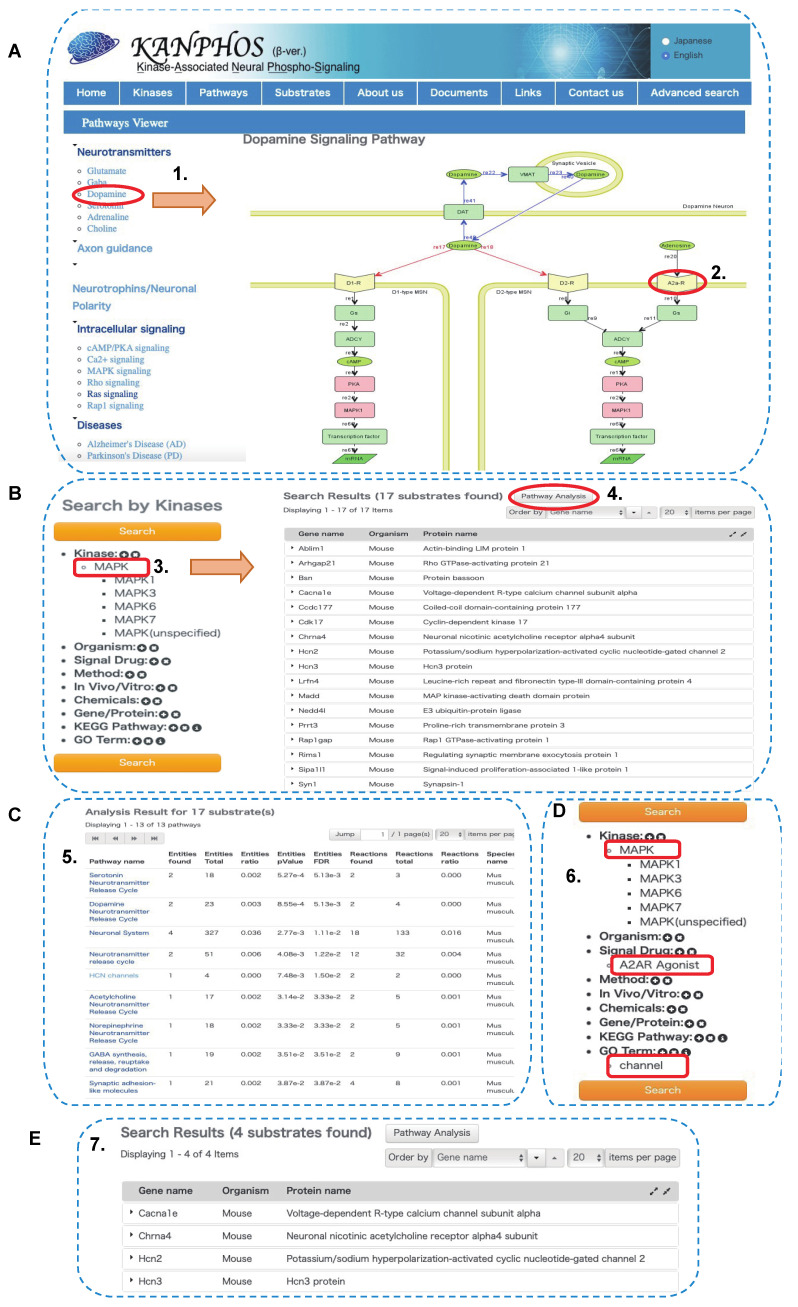
A case study of the KANPHOS revealed that phosphorylation of HCN and calcium channel were involved in the MEK-MAPK pathway downstream of A2AR. (**A**) Pathway viewer window showed dopamine signaling pathway in D1R-MSN and D2R-MSN; (**B**) List of MAPK candidate substrates downstream of A2AR in KANPHOS; (**C**) Results of pathway analysis using KANPHOS; (**D**) Advanced search option for the identification of channel protein in the MEK-MAPK pathway downstream of A2AR; (**E**) List of channel protein involved in the MEK-MAPK pathway downstream of A2AR. Here, number (1–7) indicate the workflow step by step, red circle and red rectangle represent the selected search options, and arrow indicates result output after each selection.

Protein phosphorylation orchestrates neurotransmitter release, neuronal polarity, and neuronal signaling pathways and is involved in the pathogenesis of many neurological diseases; thus, pathway analysis using the KANPHOS database could provide a means to understand how phosphorylation events can regulate neuronal signaling events. In the previous section, we identified MAPK candidate substrates. Here, we propose a workflow for analyzing MAPK phosphoproteomics data using KANPHOS pathway analysis as a model study to identify novel phosphoproteins downstream of the MEK-MAPK pathway in A2AR (Figure 7). First, click on the “Pathways” icon on the home page, which opens the pathway viewer window. Here, we can find five main search options on the left frame: 1. neurotransmitter, 2. axon guidance, 3. neuronal polarity, 4. intracellular signaling, and 5. neuronal diseases. Additionally, each option is composed of several other search options that users can select according to their interests. As we wanted to focus on A2AR signaling, we began with the neurotransmitter section by choosing dopamine, which opened the pathway viewer image of dopamine signaling in dopamine neurons (Figure 7A). Effector molecules are designated as green, and kinases are designated as pink. Clicking on a specific kinase opens the detailed view of its substrate list along with all phosphorylation information. MAPK was selected from the D2R-MSN signaling pathway, and 17 phosphoproteins were found to be candidate substrates of MAPK downstream of A2AR signaling (Figure 7B). To obtain more information on the identified candidate substrates, the user can perform pathway analysis by clicking on the upper right corner of the substrate list to reveal the associated Gene Ontology terms. All the identified pathways related to the candidate substrates will appear in a tabular format according to their *p*-value (Figure 7C). Here, we found several phosphoproteins that are involved in regulating hyperpolarization-activated cyclic-nucleotide gated (HCN) channels. To further find which phosphoproteins are involved in this channel regulation process downstream of A2AR, next we go to the advance search option and from which we select Kinase (MAPK), signal drug (A2AR Agonist) and GO term (Channel) (Figure 7D). After choosing this option, the search can be conducted and results will appear including phosphoprotein name. Here, we identified, HCN channels (Hcn2, Hcn3), and voltage-dependent R-type Calcium voltage-gated channel Alpha 1E subunit (Cacna1e) (Figure 7E). Previously, it was reported that MAPK was involved in regulating HCN channel [49,50] and calcium channel function [51]. Thus, KANPHOS pathway analysis can help researchers to explore further associations between MAPK and HCN and calcium channel properties downstream of A2AR signaling. The bottom left corner of the pathway search window displays specific sections for neuronal diseases, e.g., Alzheimer’s disease, Parkinson’s disease, and schizophrenia. By clicking on a specific disease, a KANPHOS pathway search can also be initiated to identify the relationships between the neurological disease and phosphorylation-dependent signaling events (Figure S3). In the pathway analysis of KANPHOS, a search for Alzheimer’s disease pathways reveals that CDK5 and GSK3*β* are important kinases involved in the pathogenesis of Alzheimer’s disease. Currently, 208 and 128 phosphoproteins are registered in KANPHOS as candidate substrates for CDK5 and GSK3*β*, respectively. For example, in these lists, CRMP2 (collapsin response mediator protein 2; also known as dihydropyrimidinase-like protein-2, DRP2 or DPYSL2) is an abundant protein in the brain that regulates axonal growth and neuronal polarity, is phosphorylated by CDK5 at S522 and GSK3*β* at T509, T514, and S518, and is involved in the pathogenesis of Alzheimer’s disease [52,53,54]. These phosphorylations are abnormally high in the brains of Alzheimer’s disease patients [55]. Thus, using KANPHOS pathway analysis, it is possible to search for kinases and their substrates involved in Alzheimer’s disease, which is useful in the search for new drug targets for the treatment of Alzheimer’s disease.

## 4. Discussion

Protein phosphorylation is one of several general mechanisms that regulates the function of proteins and signal transduction in the cell, which regulates many biological processes, including cell proliferation, growth, differentiation, and metabolism. To understand the biological role of phosphorylation, it is necessary to characterize upstream signal transduction, including the kinases responsible for phosphorylation and the functional changes in downstream molecules. Phosphorylation signaling cascades vary among tissues, and completely different cellular reactions can result from the same protein phosphorylation event in different tissues due to slight differences in the cascade components. Our KANPHOS database is designed to refer not only to phosphorylation and the responsible kinases but also to related diseases, genetic polymorphisms, and knockout mice. By examining these associated data and pathway analysis of the protein phosphorylation selected in the KANPHOS search results, the signaling cascades participating in the phosphorylation of the target protein can be predicted. Compared to the other conventional databases, brain-specific, signal transduction and kinase-oriented phosphorylation data collection is unique to KANPHOS, and would provide high quality data with good usability for neuroscientists.

Our current phosphorylation data are derived mainly from the mouse striatum and from cell lines such as HeLa cells, but other tissues such as the mouse forebrain, hippocampus, liver, muscle, and cancer cell lines could be further targets of our KISS/KIOSS phosphoproteomics research. With recent improvements to the CRISPR system, kinases can be easily knocked out in various cell lines. Our in vivo protein phosphorylation screening method, KIOSS, can be used to monitor phosphorylation-dependent signaling cascades that completely lack the target kinases. However, there remain several limitations for improving in KANPHOS. The tissues and cells deposited in the database are mainly brain and neuronal cells. We need to obtain and process phosphoproteomic data from other repositories, along with empirically examining other tissues and cell lines to produce additional tissues/cell-specific phosphoproteomic profiles, to add to our database. The number of target kinases in KIOSS is limited because we need to establish effective agonist/antagonist treatment conditions for each kinase, but knockdown of each kinase by improved gene targeting methods will help us to systematically perform KIOSS analysis on kinases of interest. As a future perspective, we will continuously update KANPHOS by incorporating phosphoproteomics data based on our experimental results and links with other databases. We are preparing the data of phosphorylation-dependent signaling by several neurotransmitters in the specific brain regions, and mounting search options to narrow down the result by brain regions as the next step.

Protein kinases such as Pak3, Limk1, ERK, and RSK2 have been implicated in intellectual disability [56,57]. However, the mechanisms by which these protein kinases are involved in intellectual disability are largely unknown. Neurodevelopment is thought to be involved in the pathogenesis of psychiatric disorders such as ASD, schizophrenia, and bipolar disorder, but the causes and etiology of these diseases are unknown. For schizophrenia, the dopamine hypothesis has been proposed because D2R antagonists are widely used as therapeutic agents for schizophrenia [58]. The glutamate hypothesis has also been proposed, since long-term administration of phencyclidine, an NMDA-R antagonist, causes schizophrenia-like symptoms [59]. D2R antagonist activates the adenosine-MAPK pathway [32]. This database will be useful to understand the pathogenesis of neurodevelopmental disorders, including intellectual disability and schizophrenia, to understand the mechanism of action of D2R antagonists and to develop new therapeutic drugs.

## Figures and Tables

**Table 1:** Statistics of the KANPHOS database.

Method/Stimulation	Kinase	No. of Phos. Site
KIOSS (1433z)/Forskolin	PKA, MAPK and other	278
KIOSS (1433z)/OA&U0126	MAPK and other	324
KIOSS (1433z)/D1R Agonist	PKA, MAPK and other	185
KIOSS (1433z)/A2AR Agonist	PKA, MAPK and other	62
KISS	CaMKI, CDK5, FYN, LYN, MAPK1, PAK7, PKA, PKN, ROCK2	3597
Protoarray	AKT1, CaMK2A, CDK5, LRRK2, LYN, MAPK1, PRKCA, PRKACA, ROCK2	1782
Outsource	AurA, AurB, CaMKI, CaMKII, CaMK4, CDK5, GSK3B, LYN, MAPK, MARK1, PKA, AMPK, PRKCA, PRKCE, ROCK, STK11(LKB1)	3736

**Table 2:** List of phosphoproteins along with phosphorylation site downstream of adenosine (A2AR) signaling.

Motif	Identified Phosphoprotein and Phosphorylation Site					
**PKA**	Arhgap21	Arhgap21	Arhgap21	Arhgap23	Brsk1	Brsk2
	S472	S851	S875	S401	S324	S383
	Bsn	Bsn	Cacna1b	Ccdc177	Ccny	Cdk17
	S1987	S2860	S2219	S303	S301	S180
	Chrna4	Fam126b	Gpr75	Hcn2	Kcnh7	Kif21b
	S540	S521	S317	S840	S174	S1150
	Madd	Mark1	Nedd4l	Nf1	Prickle2	Prrt3
	S1038	S394	T353	S2524	S752	S846
	Rap1gap	Rims1	Sik3	Syn1	Taf4b	Ttc32
	S245	S887	S454	S427	S707	S24
**MAPK**	Ablim1	Arhgap21	Bsn	Cacna1e	Ccdc177	Cdk17
	S89	S42	Y3020	S791	S308	T11
	Chrna4	Chrna4	Hcn3	Lrfn4	Madd	Madd
	S540	S543	S633	S627	S1038	T1045
	Nedd4l	Prrt3	Rap1gap	Sipa1l1	Sorbs2	Spata2
	S329	S834	S213	S1528	S944	S247
	Syn1					
	S427					
**Others**	Arhgap21	Atp6v1h	Cep170	Gpr75	Kcnh2	Khnyn
	S856	Y125	S353	T313	S322	S43
	Madd	Mapk8	Rsl1d1	Rsl1d1	Sh2d5	Ttc32
	S1089	S210	T314	S316	S126	T18

**Table 3:** List of phosphoproteins identified as a MAPK candidate substrates.

Identified MAPK Candidate Substrates List							
2010300C02Rik	Atg9a	Cdk18	Epb4.1l3	Lppr3	Osbpl6	Rasgrp2	Ssh2
Aak1	Bai1	Cdkl5	Epb49	Lrfn4	Pak7	Rem2	Stim1
Abi1	Baiap2	Cep170	Erc2	Lrrc7	Panx2	Rims1	Syn1
Abi2	Bcr	Chrna4	Etl4	Madd	Pclo	Rims2	Syn2
Agap2	Begain	Clasp2	Fam171a1	Map3k5	Pfkfb2	Rph3a	Syn3
Agfg1	Brsk1	Crtc2	Fam171a2	Map4	Pip5k1c	Rrad	Synpo
Akap6	Brsk2	Ctnnd2	Fam171b	Mark1	Pitpnm3	Sh2d5	Syt7
Als2	Bsn	Cyth3	Frmd4a	Mark3	Plekha5	Shank3	Tanc2
Ampd2	C17orf59	Dab2ip	Gab2	Mast1	Plekho2	Shisa7	Tpd52
Ank2	C2cd4c	Dennd1a	Git1	Mast3	Pola1	Sipa1l1	Tpd52l1
Ankrd34a	Cacna1b	Dennd4c	Gm1568	Mbp	Ppfia2	Sipa1l2	Tsc2
Ankrd34b	Cacna1e	Dgkq	Gm15800	Mink1	Ppfia3	Slc4a4	Uhrf1bp1l
Anks1b	Camk2g	Dlgap2	Gpr158	Mllt4	Ppfia4	Sorbs1	Ulk1
Arhgap21	Camkk1	Dlgap3	Hcn2	Mprip	Prrt3	Sorbs2	Ulk2
Arhgap23	Camkk2	Dos	Hcn3	Nav1	Psd	Spata2	Usp31
Arhgap32	Camsap2	Dpysl2	Iqsec1	Nav3	Psd3	Specc1	Usp8
Arhgap39	Caskin1	Dpysl5	Kcnb1	Ndel1	Rab11fip2	Speg	Wdr47
Arhgef2	Ccdc22	Dstn	Kiaa0284	Nelf	Ralgapa1	Srcin1	
Arhgef6	Ccny	Dtna	Kiaa0528	Nhsl2	Rap1gap	Srgap3	
Atat1	Ccnyl1	Eif3d	Kiaa1211	Nyap2	Rapgef2	Ssfa2	
**Total 157**							

## Data Availability

The data presented in this study are available in Amano et al., 2015 [25] and Nagai et al., 2016 [26], and the other data are openly available online: “https://kanphos.neuroinf.jp” (accessed on 1 December 2021).

## Supplementary Materials

The following are available online at https://www.mdpi.com/article/10.3390/cells11010047/s1: Figure S1: Details information and search options on result window of a phosphoprotein. Figure S2: Advance search options in KANPHOS. Figure S3: Identification of signaling network and phosphoproteins involvement in Alzheimer’s disease by KANPHOS pathway analysis. Table S1: List of external databases associated with KANPHOS database.
