# Supplementary material for: KANPHOS: A Database of Kinase-Associated Neural Protein Phosphorylation in the Brain
Source: Cells. 2021 Dec 24;11(1):47. doi: 10.3390/cells11010047 (PMC8750479; doi:10.3390/cells11010047)
Supplement: Supplementary file 1 [file cells-11-00047-s001.zip › cells-1515978-supplementary.pdf]

**Figure S1:**

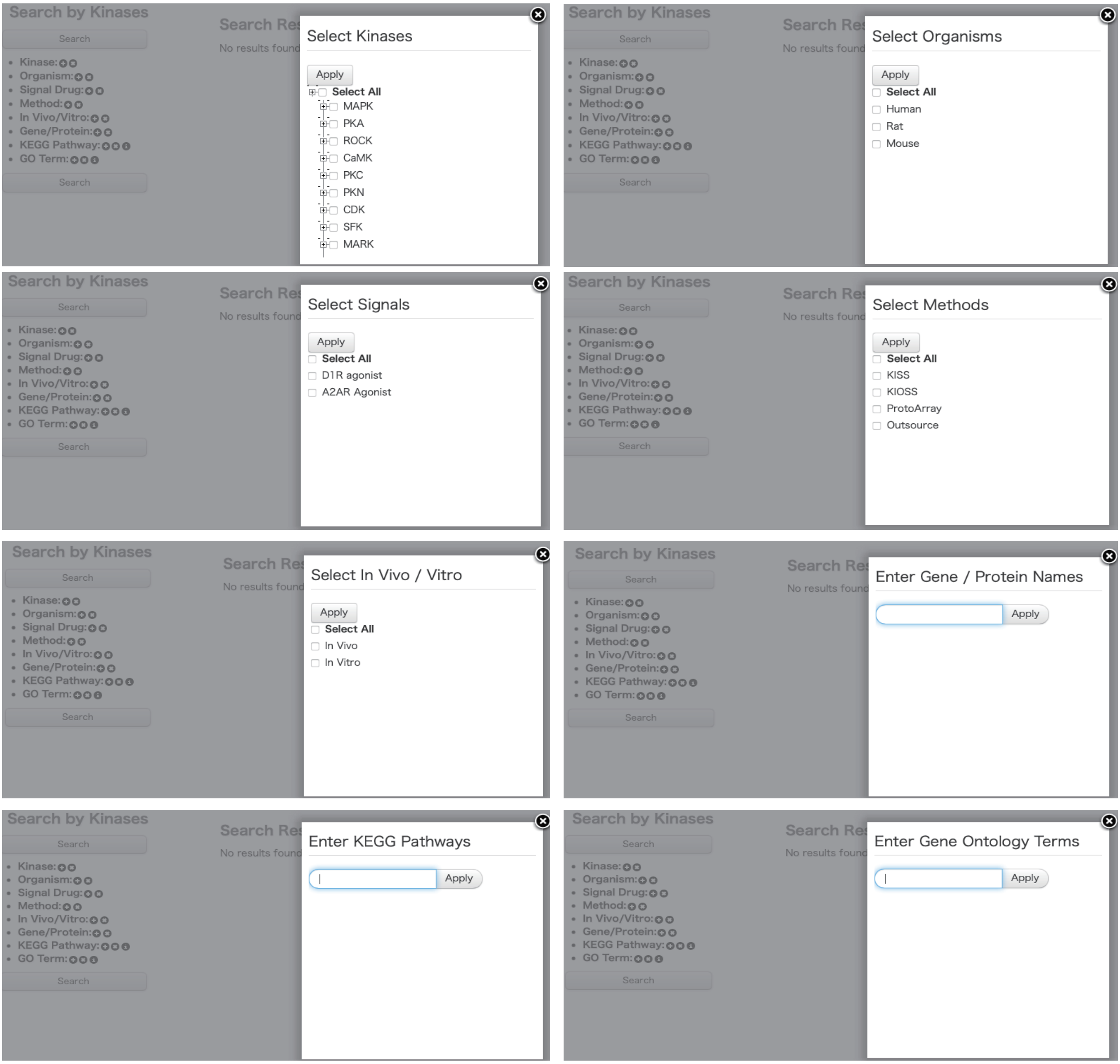
Search by Kinases
Search
Kinase:
Organism:
Signal Drug:
Method:
In Vivo/Vitro:
Gene/Protein:
KEGG Pathway:
GO Term:
Search
Search Res
No results found
Select Kinases
Apply
Select All
MAPK
PKA
ROCK
CaMK
PKC
PKN
CDK
SFK
MARK
Select Organisms
Apply
Select All
Human
Rat
Mouse
Select Signals
Apply
Select All
D1R agonist
A2AR Agonist
Select Methods
Apply
Select All
KISS
KIOSS
ProtoArray
Outsource
Select In Vivo / Vitro
Apply
Select All
In Vivo
In Vitro
Enter Gene / Protein Names
Apply
Enter KEGG Pathways
Apply
Enter Gene Ontology Terms
Apply

**Figure S2:**

Chrna4 Mouse Neuronal nicotinic acetylcholine receptor alpha4 subunit

Substrate Information

| | |
|---|---|
| **Organism** | Mouse (Mus musculus) |
| **Uniprot ID** | Q53YK0_MOUSE |
| **Accession #** | Q53YK0 |
| **Protein names** | Neuronal nicotinic acetylcholine receptor alpha4 subunit |
| **Gene names** | Chrna4 |
| **Description** | N/A |
| **Links** | Search Kinases of Chrna4 (Mouse) |

KEGG Pathways (4)

Chemical carcinogenesis
Cholinergic synapse
Neuroactive ligand
Nicotine addiction

Gene Ontology Terms (4)

acetylcholine-gated cation-selective channel activity
integral component of membrane
postsynaptic membrane
transmembrane signaling receptor activity

Phosphorylation Site Information

| Kinase | Residue | Sequence | Method | | Signal | Chemicals |
|---|---|---|---|---|---|---|
| 0000PKA | S540 | VSDQTSPCKCTCKEPSPVSPITVLKAGGTKA | KIOSS (14-3-3z) | in Vivo | CGS21680 (A2AR Agonist/Activator) | |

**Figure S3**

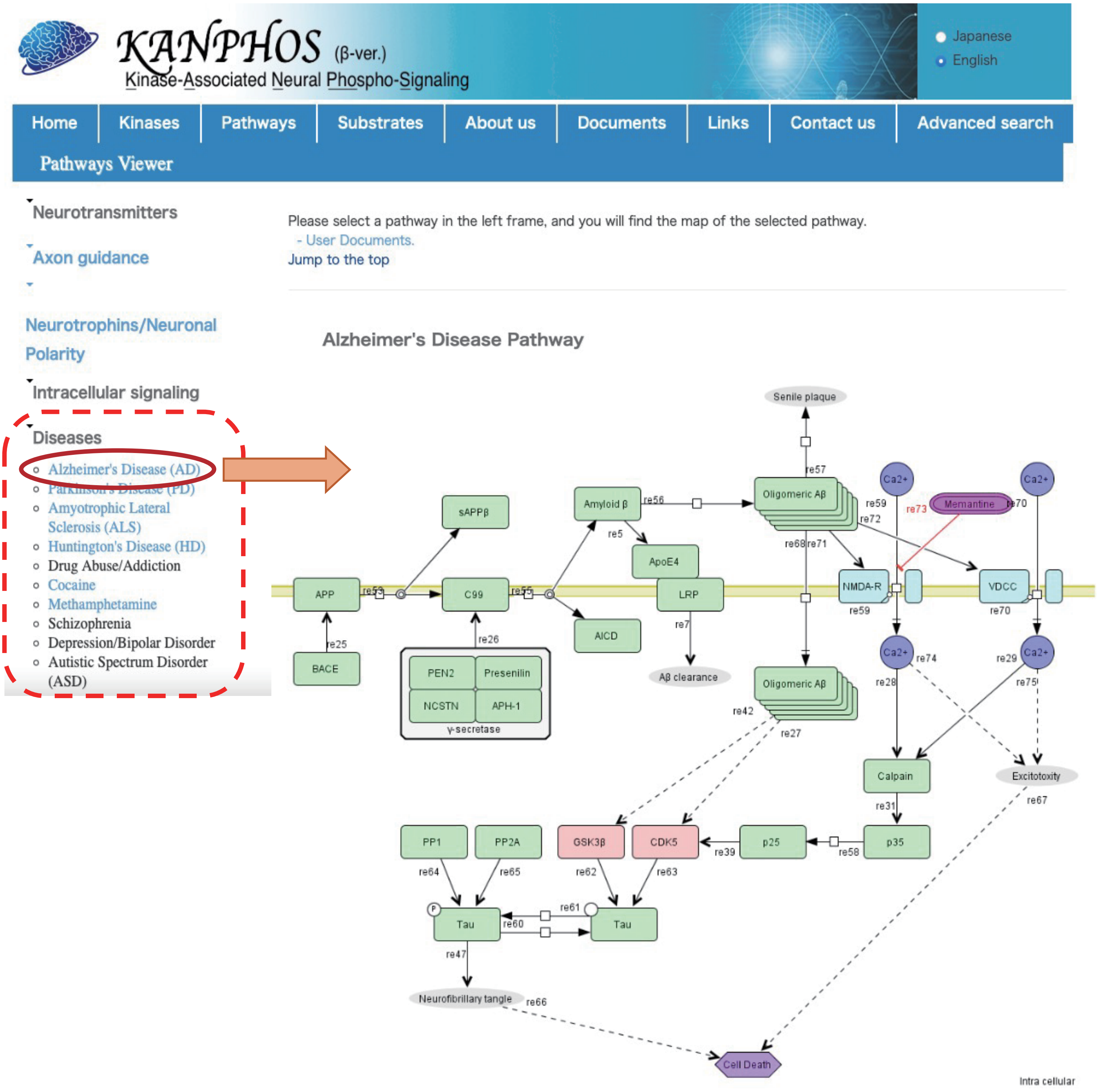
KANPHOS (β-ver.)
Kinase-Associated Neural Phospho-Signaling
Japanese
English
Home
Kinases
Pathways
Substrates
About us
Documents
Links
Contact us
Advanced search
Pathways Viewer
Neurotransmitters
Axon guidance
Neurotrophins/Neuronal Polarity
Intracellular signaling
Diseases
Alzheimer's Disease (AD)
Parkinson's Disease (PD)
Amyotrophic Lateral Sclerosis (ALS)
Huntington's Disease (HD)
Drug Abuse/Addiction
Cocaine
Methamphetamine
Schizophrenia
Depression/Bipolar Disorder
Autistic Spectrum Disorder (ASD)
Please select a pathway in the left frame, and you will find the map of the selected pathway.
- User Documents.
Jump to the top
Alzheimer's Disease Pathway
Senile plaque
Oligomeric Aβ
Amyloid β
sAPPβ
ApoE4
APP
C99
LRP
NMDA-R
VDCC
Ca2+
Memantine
BACE
AICD
PEN2
Presenilin
NCSTN
APH-1
γ-secretase
Aβ clearance
Calpain
Excitotoxity
PP1
PP2A
GSK3β
CDK5
p25
p35
Tau
Neurofibrillary tangle
Cell Death
Intra cellular

**Table S1:** List of external database associated with KANPHOS database

| Database Name | URL |
|---|---|
| Allen Brain Atlas | http://portal.brain-map.org |
| DISEASES: Disease-gene associations mined from literature | http://diseases.jensenlab.org |
| GeneCards | http://www.genecards.org |
| GO (Gene Ontology) database | http://geneontology.org |
| H-Invitational Database (H-InvDB) | http://www.h-invitational.jp |
| HGNC (HUGO Gene Nomenclature Committee) database | http://www.genenames.org |
| HomoloGene | http://www.ncbi.nlm.nih.gov/homologene |
| KEGG PATHWAY database | http://www.genome.jp/kegg/pathway.html |
| MalaCards: human disease database | http://www.malacards.org |
| Mouse Genome Informatics (MGI) | http://www.informatics.jax.org |
| Online Mendelian Inheritance in Man (OMIM) | http://www.omim.org |
| PhosphoSitePlus | http://www.phosphosite.org |
| Rat Genome Database (RGD) http://rgd.mcw.edu | http://rgd.mcw.edu |
| SZGene (SchizophreniaGene) http://www.szgene.org | http://www.szgene.org |
| UniProt Knowledgebase (UniProtKB) | http://www.uniprot.org |
